# LIFETIME EXERCISE ATTENUATES AGE- AND WESTERN DIET-RELATED DECLINES IN PHYSICAL FUNCTION IN MICE

**DOI:** 10.1093/geroni/igz038.391

**Published:** 2019-11-08

**Authors:** Zachary S Clayton, Rachel A Gioscia-Ryan, Jamie N Justice, Kara Lubieniecki, Matthew Rossman, Melanie Zigler, Douglas Seals

**Affiliations:** 1 University of Colorado Boulder, Boulder, Colorado, United States; 2 Wake Forest School of Medicine, Winston-Salem, North Carolina, United States

## Abstract

Aging is associated with progressive declines in physical function. However, it is unknown if consumption of a western-style diet (WD; high-fat and sucrose, low fiber), compared with a non-WD (healthy diet), accelerates declines in physical function over the adult lifespan, and whether regular voluntary exercise attenuates age- and WD-associated declines in function. To determine this, we studied 4 cohorts of male C57BL/6 mice that consumed either normal chow [NC] or WD with or without access to voluntary running [VR] wheels beginning at 3 mo of age and assessed strength (grip strength normalized to body mass) and endurance (rota-rod distance) every 3 mo throughout life. WD decreased average lifespan by 30% (WD: 18.6±0.5 vs. NC: 26.7±0.8 mo); therefore, function was compared from 3-18 mo of age in all groups. Age-related declines (% change over 3-18 mo) in physical function were accelerated by WD (strength: WD -61.2±10.1%, NC -43.2±10.2%; endurance: WD -97.4±5.1%, NC -65.1±6.3%; all p<0.05 WD vs. NC). VR attenuated declines in physical function within the same diet group (strength: WDVR -34.7±5.1%, NCVR -18.6±5.2%; endurance: WDVR -48.5±5.2%, NCVR -41.4±4.7%; all p<0.05 versus same diet non-VR group). These unique data obtained from a lifelong study of aging in mice, indicate that: 1) consuming a WD reduces lifespan and accelerates age-related declines in physical function by 40-50% vs. a non-WD; regular voluntary exercise (wheel running) prevents this effect of WD on physical function; and 2) regular voluntary exercise also attenuates the age-associated decline in physical function by ~60-130% when consuming a healthy diet.

